# Supplementary material for: Measuring speaker–listener neural coupling with functional near infrared spectroscopy
Source: Sci Rep. 2017 Feb 27;7:43293. doi: 10.1038/srep43293 (PMC5327440; doi:10.1038/srep43293)
Supplement: Supplementary Information [file srep43293-s1.pdf]

Supplementary Information

for

Measuring speaker–listener neural coupling with functional near infrared spectroscopy

Yichuan Liu[1], Elise A. Piazza[2], Erez Simony[2], Patricia A. Shewokis[1,4,5], Banu Onaral[1], Uri Hasson[2,5], Hasan Ayaz[1,6,7, *]

[1] School of Biomedical Engineering, Science & Health Systems, Drexel University, Philadelphia, PA, USA

[2] Princeton Neuroscience Institute, Princeton University, Princeton, NJ, USA

[3] Nutrition Sciences Department, College of Nursing and Health Professions, Drexel University, Philadelphia PA, USA

[4] Department of Surgery, College of Medicine, Drexel University, Philadelphia, PA, USA

[5] Department of Psychology, Princeton University, Princeton, NJ, USA

[6] Department of Family and Community Health, University of Pennsylvania, Philadelphia, PA, USA

[7] Division of General Pediatrics, Children's Hospital of Philadelphia, Philadelphia, PA, USA

* Corresponding Author: hasan.ayaz@drexel.edu

Table S1 Optode locations in automatic anatomical labeling (AAL) [1,2]

AAL Area	Optodes	
	Left hemisphere	Right hemisphere
Angular gyrus	22, 24, 25, 27	30, 32, 33, 35
Superior frontal gyrus, medial orbital (Frontal_Med_Orb)	6, 8	10
Middle frontal gyrus (Frontal_Mid)	1, 3, 5	13, 15
Middle frontal gyrus, orbital part (Frontal_Mid_Orb)	2, 3, 4, 6	12, 14, 16
Superior frontal gyrus, dorsolateral (Frontal_Sup)	3, 4, 5, 6, 7	11, 12, 13
Superior frontal gyrus, medial (Frontal_Sup_Medial)	7	9
Superior frontal gyrus, orbital part (Frontal_Sup_Orb)	4, 6, 8	10, 12, 14
Superior occipital gyrus	28	35, 38
Inferior parietal, but supramarginal and angular gyri (Parietal_Inf)	17, 19, 22, 24, 25, 28	29, 30, 32, 35
Superior parietal gyrus (Parietal_Sup)	18, 20, 21, 22, 23, 25, 26, 28	29, 31, 32, 34, 35, 36, 37, 38, 39, 40
Postcentral gyrus	17, 18, 19	31, 36
Precuneus	21, 26	39, 40

Table S2 Optode locations in Brodmann areas[1]

Brodmann area	Optodes	
	Left hemisphere	Right hemisphere
Area 1	17, 18, 19	31, 36
Area 10	1, 2, 3, 4, 5, 6, 7, 8	9, 10, 11, 12, 13, 14, 15
Area 11	3, 4, 5, 6, 8	10, 11, 12, 13, 14
Area 19	27, 28	33, 38
Area 2	17, 19	29, 31
Area 3	17	31, 36
Area 39	22, 24, 25, 27	30, 32, 33, 35
Area 40	17, 19, 20, 22, 24	29, 30, 32
Area 46	1, 2, 4	14, 15, 16
Area 47	2, 4	14, 16
Area 5	18, 21	36, 39
Area 7	18, 20, 21, 22, 23, 25, 26	32, 34, 35, 36, 37, 38, 39

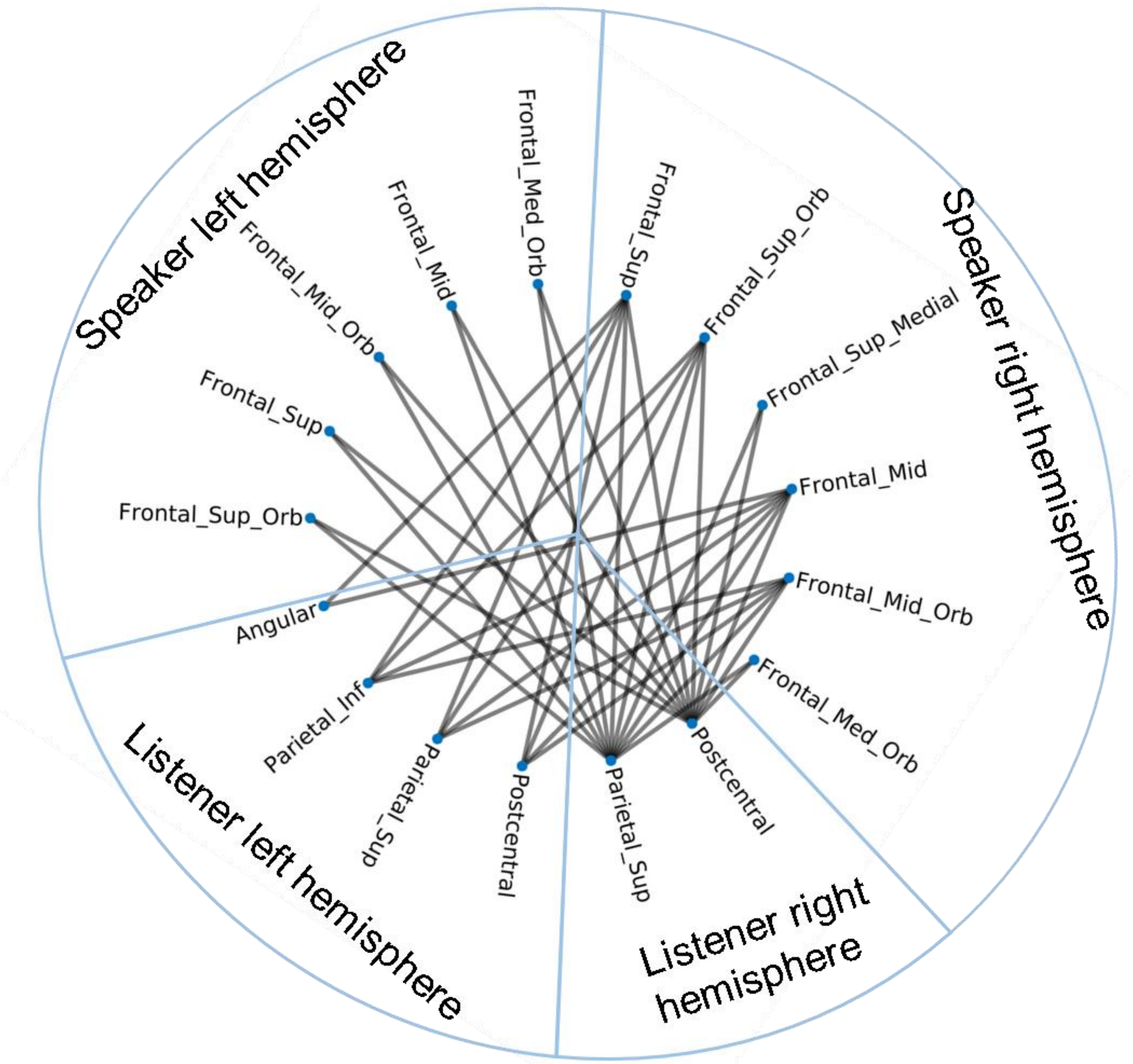

Figure S1 Speaker-listener coupling for E1 story at 5 sec lag (speaker leads).

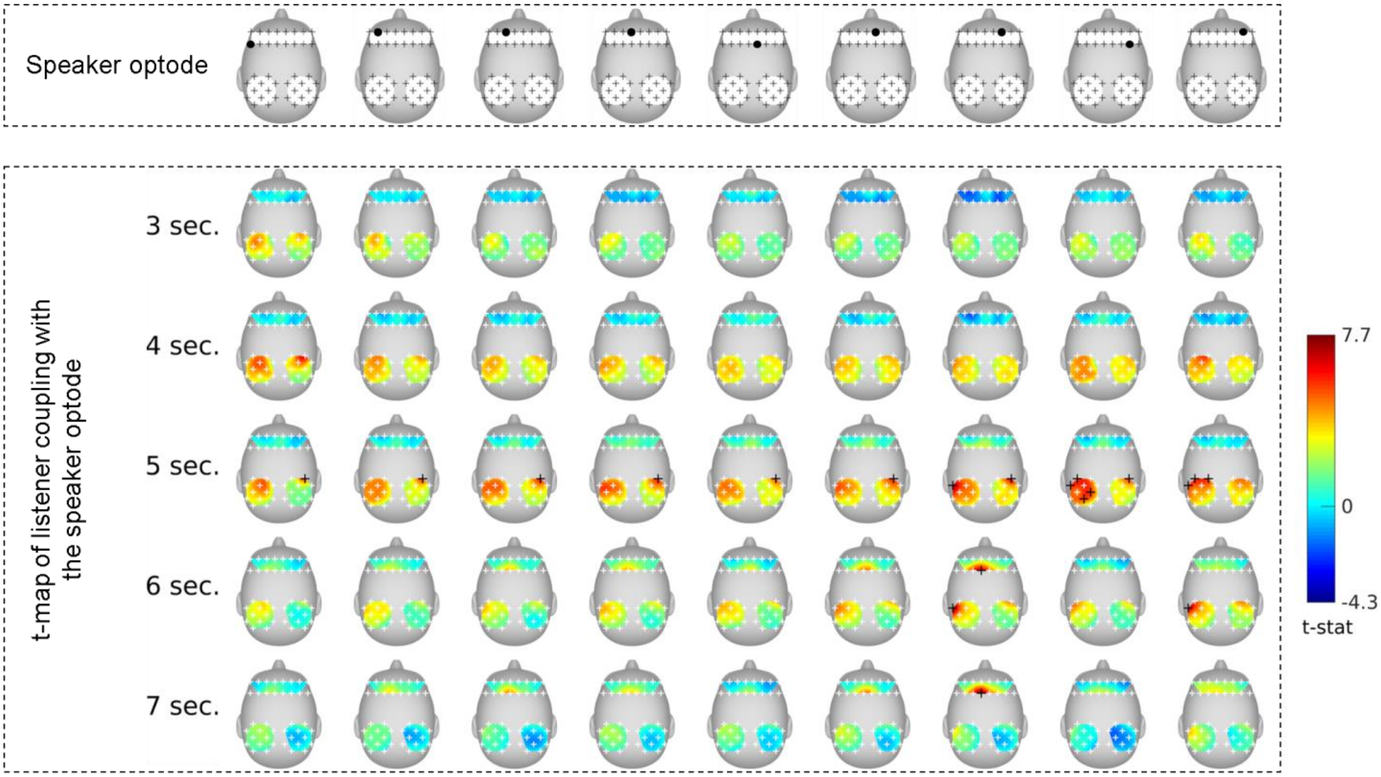

Figure S2 Speaker-listener non-homologous coupling t-map for E1 story. Each row shows the couplings at a time lag (speaker leads). Each column shows t-maps of listener coupling with a specific speaker optode. Black "+" sign on the t-maps represents significant optodes (FDR q < 0.01).

References

1 Tsuzuki, D. *et al.* Virtual spatial registration of stand-alone fNIRS data to MNI space. *NeuroImage* **34**, 1506-1518, doi:http://dx.doi.org/10.1016/j.neuroimage.2006.10.043 (2007).

2 Tzourio-Mazoyer, N. *et al.* Automated Anatomical Labeling of Activations in SPM Using a Macroscopic Anatomical Parcellation of the MNI MRI Single-Subject Brain. *NeuroImage* **15**, 273-289, doi:http://dx.doi.org/10.1006/nimg.2001.0978 (2002).
